# Downregulated miR-204 Promotes Skeletal Muscle Regeneration

**DOI:** 10.1155/2020/3183296

**Published:** 2020-11-17

**Authors:** Ya Tan, Linyuan Shen, Mailin Gan, Yuan Fan, Xiao Cheng, Ting Zheng, Lili Niu, Lei Chen, Dongmei Jiang, Xuewei Li, Shunhua Zhang, Li Zhu

**Affiliations:** ^1^College of Animal Science and Technology, Sichuan Agricultural University, Chengdu, Sichuan 611130, China; ^2^Farm Animal Genetic Resources Exploration and Innovation Key Laboratory of Sichuan Province, Sichuan Agricultural University, Chengdu, Sichuan 611130, China; ^3^Institute of Animal Husbandry and Veterinary, Guizhou Academy of Agricultural Science, Guiyang, Guizhou 550005, China

## Abstract

Skeletal muscle is the most abundant and a highly plastic tissue of the mammals, especially when it comes to regenerate after trauma, but there is limited information about the mechanism of muscle repair and its regeneration. In the present study, we found that miR-204 is downregulated after skeletal muscle injury. In vitro experiments showed that over-expression of miR-204 by transfecting with miR-204 mimics suppressed C2C12 cell proliferation, migration, and blocked subsequent differentiation, whereas inhibition of miR-204 by transfecting with miR-204 inhibitor showed the converse effects. Furthermore, through the dual luciferase reporter system, we demonstrated that miR-204 can target the 3'UTR regions of *Pax7*, *IGF1*, and *Mef2c* and inhibit their expression. Taken together, our results suggest that *Pax7*, *IGF1*, and *Mef2c* are the target genes of miR-204 in the process of myoblasts proliferation, cell migration, and differentiation, respectively, and may contribute to mouse skeletal muscle regeneration. Our results may provide new ideas and references for the skeletal muscle study and may also provide therapeutic strategies of skeletal muscle injury.

## 1. Introduction

Tissue and organ regeneration is common in metazoans, and it is puzzling that regenerative capacity has evolved losses in higher animals [1]. Skeletal muscle is one of the mammalian tissues with a strong regenerative capacity [2]. Skeletal muscle represents about 30~40% of the total body mass in animals [3]. It is not only a motor organ but also an important energy metabolism and endocrine organ. The ability of the skeletal muscle to regenerate depends on the muscle satellite cells, which are located between the sarcolemma and the basal layer of the skeletal muscle fibers [4]. Usually, the muscle satellite cells remain static, can be activated by being injured, and then change from a static state to a myogenic process. Some of the muscle satellite cells that have proliferated by asymmetric division migrate to the damaged part to differentiate into the damaged part of the myotube repair, and the other part returns to the quiescent state, refilling the satellite cell pool [5].

The activation [6], proliferation [7], migration [8], and differentiation [9] of satellite cells are the main processes of muscle regeneration, and a large number of genes have been found to be involved in the process of skeletal muscle regeneration [10]. C2C12 myoblasts, a cell line that is derived from the satellite cells isolated from murine skeletal muscle, are the best immortalized model to study the reserved myoblast function of satellite cells [11, 12]. miRNAs are a class of noncoding RNAs of about 22 nt in length that function by inhibiting their target gene expression. With the deepening of miRNA research, some miRNAs have also been found to be involved in the regulation of skeletal muscle regeneration [13]. In recent years, miR-204 has been found to be associated with many biological processes, such as cancer [14], inflammation [15], and lipid metabolism [16], but the role of miR-204 in skeletal muscle regeneration is not fully understood.

In this study, we constructed an acute muscle injury model of skeletal muscle in mice, using cardiotoxin (CTX) to detect the expression level of miR-204 during muscle regeneration. Additionally, C2C12 myoblasts were used as an *in vitro* model to investigate the function of miR-204 in the proliferation, migration, and differentiation of C2C12 myoblasts by overexpression or inhibition of miR-204. All the results of this study may be able to elucidate the effects of miR-204 on skeletal muscle regeneration and provide valuable information for the diagnosis and treatment of muscle damage.

## 2. Materials and Methods

### 2.1. Animals and Treatment

Animals were housed in the Animal Resources under controlled temperature (22°C ± 3°C) for a natural light cycle and allowed ad libitum to feed and water. ICR mice (♀, 8 weeks) were purchased from the Chengdu Da Shuo Experimental Animal Co., Ltd. (Chengdu, China). A model of anterior tibial muscle (TA) injury was induced by injection of cytotoxin (CTX, Sigma, USA) with the dosage of 10*μ*M [17]. All the experimental procedures and sample collection were approved by the Institutional Animal Care and Use Committee of the College of Animal Science and Technology of Sichuan Agricultural University, Sichuan, China, under permit No. DKY-2018102010.

### 2.2. Tissue Section

The obtained mouse TA was fixated with 4% paraformaldehyde; then, the tissues were dehydrated using ethanol, infiltrated, and embedded using paraffin; and finally, the tissue sections (4-5 *μ*M thickness) were stained with hematoxylin and eosin (H&E) for histological analysis.

### 2.3. Cell Culture and Treatment

C2C12 myoblasts purchased from Stem Cell Bank, Chinese Academy of Science, were cultured at 37°C in a CO_2_ incubator (5% CO_2_). The C2C12 cells were maintained in Dulbecco's modified Eagle's medium (DMEM, Gibco, Carlsbad, CA, USA) and supplemented with 10% fetal bovine serum. When the C2C12 myoblasts reached 80% confluence, the cells were moved to differentiation medium (DM) which consist of DMEM and 2% horse serum (Gibco). miR-204 mimic, inhibitor, and negative control (Ribobio, Guangzhou, China) were transfected into the C2C12 myoblasts using Lipofectamine 3000 (Invitrogen, USA) [18].

### 2.4. Cell Proliferation Assay

Cell proliferation assays refer to previous studies [19]. The CCK-8 kit (Beyotime, Shanghai, China) was used at 24 hours, 48 hours, and 96 hours after transfection according to the manufacturer's protocol. Forty-eight hours after transfection, 10*μ*M EdU (Ribobio, Guizhou, China) was added to the growth medium, and the cells were cultured for 2 h. Subsequently, the EdU staining was performed according to the manufacturer's protocol.

### 2.5. Scratch Test

The C2C12 myoblasts were manually scraped off with a p200 pipette tip to create scratches [20, 21]. After the scratch defect was made, the cells were washed in PBS and cultured in DMEM. The C2C12 myoblasts were then incubated at 37°C for 0, 12, or 24 hours; observed; and photographed.

### 2.6. Immunocytochemistry

The differentiated C2C12 myoblasts were washed with PBS, fixed with 4% paraformaldehyde for 20 minutes, and permeabilized with 0.2% Triton X-100 for 10 minutes at room temperature (22°C ± 3°C). Then, nonspecific antibody binding was blocked with 5% goat serum (Bioss, Beijing, China) for 2 h and subsequently incubated with anti-MyHC (ab51263, Abcam, USA) overnight at 4°C. The next day, the C2C12 myoblasts were incubated with Alexa Fluor® 488 secondary antibody (ab150113; Abcam) for 1h. Finally, the cells were observed on a fluorescence microscope (Olympus, Kyoto, Japan) [22].

### 2.7. Luciferase Reporter Assay

The 3'UTR of *Pax7* (Paired box 7), *IGF1* (insulin-like growth factor 1), and *Mef2c* (myocyte enhancer factor2) (TsingKe Biotech, Chengdu, China) containing the miR-204 binding site was inserted into the psiCHECKTM-2 vector (Promega, USA), and then, the psiCHECKTM-2 vector and miR-204 mimic were cotransfected into HeLa cells (ATCC, USA) using the Lipofectamine™ 3000 reagent (Invitrogen, USA). Finally, the Firefly fluorescence and Renilla fluorescence were measured using the Dual-Glo Luciferase Assay System (Promega, Madison, USA) [23].

### 2.8. Quantitative Real-Time PCR (qRT-PCR)

qRT-PCR was performed using the SYBR® Premix Ex Taq™ kit (TaKaRa) on a CFX96 Real-Time PCR detection system (Bio-Rad, Richmond, CA, USA). The relative expression of mRNAs and miRNAs was calculated using the 2^−*ΔΔ*Ct^ method [24]. The sequences of primers for qRT-PCR are listed in Table 1.

### 2.9. Statistical Analyses

All quantitative results are presented as mean ± SD. Statistical analyses were conducted using the SPSS 20.0 software. Unpaired Student's *t*-test was performed for comparison between the treated and untreated control (NC) groups, and one-way ANOVA analysis was conducted for comparing multiple groups followed by a Dunnett's method post hoc analysis. Statistical significance was defined as *p* < 0.05.

## 3. Results

### 3.1. Downregulation of miR-204 Expression after Muscle Injury

**Figure 1:**
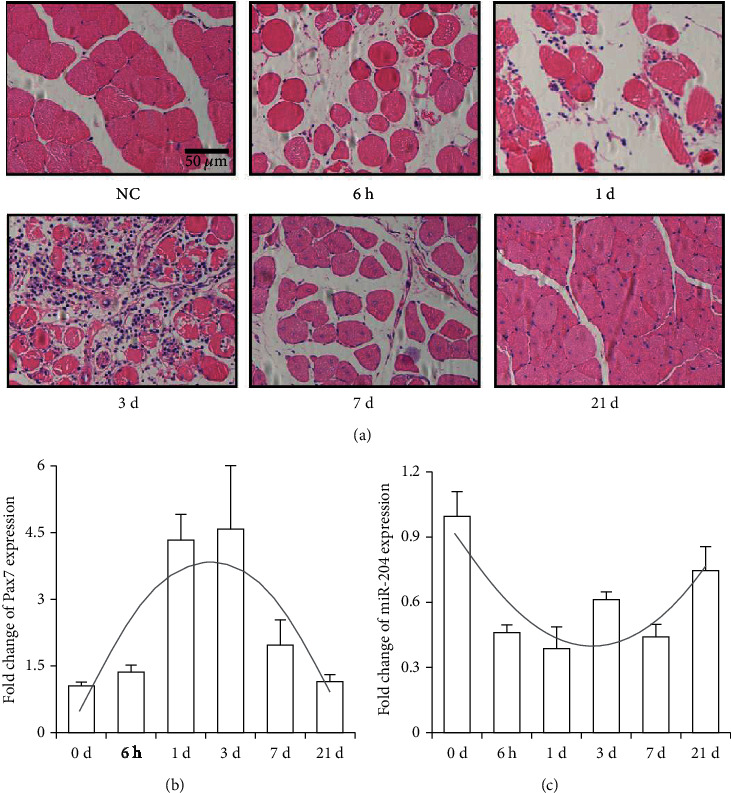
Downregulation of miR-204 expression after muscle injury. (a) HE staining of mouse TA muscle. Scale bar = 50*μ*m, *n* = 8. (b), (c) The expression of *Pax7* (Paired box 7) and miR-204 in the TA muscle of mice after injury. *n* = 3. Data are presented as means ± SD.

After CTX (cardiotoxin) injection, a mouse TA (tibialis anterior) muscle injury model was successfully constructed. The HE staining of the TA muscle showed that a large number of muscle fibers were dissolved after CTX injection for 6h, and inflammatory infiltration occurred after 1d. A large number of macrophages appeared at 3 days, and new muscle fibers were formed at 7 days. On 21d, the muscle fibers were basically repaired, but there were still a certain number of central nuclear muscle fibers (Figure 1(a)). In the process of muscle regeneration, the expression level of *Pax7*, a marker of muscle satellite cells, showed a pattern of increasing first and then decreasing (Figure 1(b)). Compared with the control group, the expression level of miR-204 showed a trend of decreasing first and then increasing and maintained a lower state throughout the muscle regeneration process (Figure 1(c)).

Pax7 Is a Target Gene of miR-204 That Regulates the Proliferation of C2C12 Myoblasts

**Figure 2:**
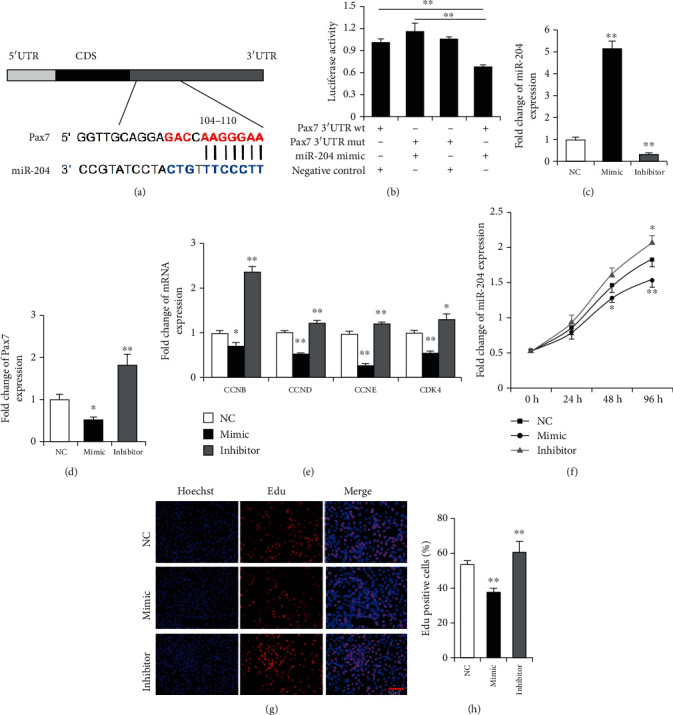
miR-204 regulates C2C12 myoblast proliferation through *Pax7*. (a) The binding site of miR-204 to *Pax7*. (b) The *Pax7* 3'UTR with wild-type (wt) or mutant type (mut) were inserted into psiCHECK™-2 vectors and cotransfected with miR-204 mimic or negative control into HeLa cells, respectively. *n* = 3. (c*–*e) The expression of miR-204 (c), *Pax7* (d), and cell division cycle genes (*CCNB* (Cyclin B), *CCND* (Cyclin D), *CCNE* (Cyclin E), *CDK4* (Cyclin-dependent kinase 4)) in the C2C12 myoblasts after transfection with miR-204 mimic or inhibitor. *n* = 3. (f) Cell counts measured using the Cell Count Kit 8 (CCK-8) method. *n* = 6. (g, h) The EdU assay was carried out after a 48h transfection. C2C12 myoblasts undergoing DNA replication were stained by EdU (red), and cell nuclei were stained with Hoechst (blue). Scale bar = 50*μ*m, *n* = 3. Data are presented as means ± SD. ^∗^*p* < 0.05, ^∗∗^*p* < 0.01.

To further investigate the function of miR-204 during skeletal muscle regeneration, the TargetScan software and sequence alignment analysis were applied in the miR-204 target finding. Then, we found miR-204 binding sites in the 3'UTR region of *Pax7* (Figure 2(a)). Subsequently, we used the dual-luciferase reporter system and found that the overexpression of miR-204 significantly inhibited the luciferase activity of the wild type of *Pax7* (Figure 2(b)). As shown in Figure 2(c), compared with the control group, the expression of miR-204 in the mimic group was elevated by approximately 5-fold. However, the expression was down to 31% in the inhibitor group. Compared with the negative control group, the expression of *Pax7*, *CCNB*, *CCND*, *CCNE*, and *CDK4* in C2C12 myoblasts was significantly downregulated by the miR-204 mimic. Meanwhile, the miR-204 inhibitor significantly promoted the expression of these genes (Figures 2(c) and 2(e)). Furthermore, the CCK8 results confirmed that miR-204 suppressed C2C12 myoblast proliferation (Figure 2(f)). In addition, these results were further verified by the EdU assay, that miR-204 mimic decreased the ratio of EdU-positive cells (Figures 2(g) and 2(h)). All these results suggest that miR-204 can inhibit C2C12 myoblast proliferation probably by targeting *Pax7*.

### 3.2. IGF1 Is a Target Gene of miR-204 That Regulates the Migration of C2C12 Myoblasts

**Figure 3:**
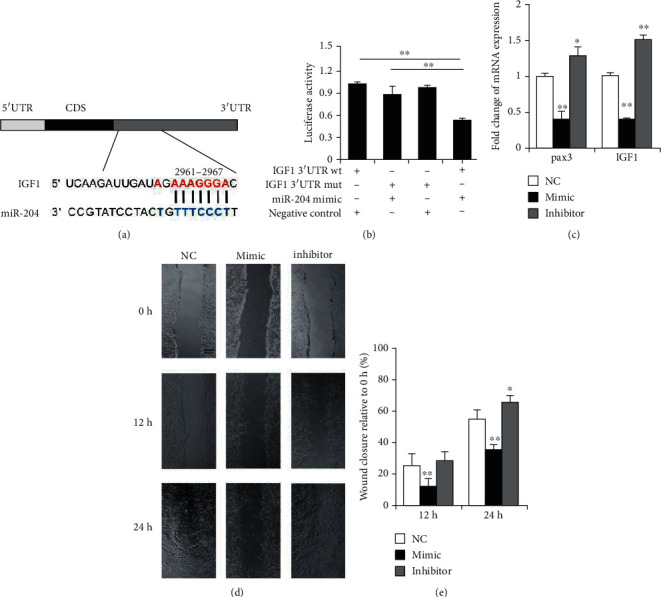
miR-204 regulates C2C12 myoblast migration through *IGF1* (insulin-like growth factor 1). (a) The binding site of miR-204 to *IGF1*. (b) The *IGF1* 3'UTR with wild-type (wt) or mutant type (mut) were inserted into the psiCHECK™-2 vectors and cotransfected with miR-204 mimic or negative control into HeLa cells, respectively. *n* = 3. (c) The expression of *Pax3* (Paired box 3) and *IGF1* in the C2C12 myoblasts after transfection with miR-204 mimic or inhibitor. *n* = 3. (d, e) Scratch test pictures of cell monolayers at time 0, 12 hours, and 24 hours following initiation of scratch defect on C2C12 myoblasts. Scale bar = 200*μ*m, *n* = 3. Data are presented as means ± SD. ^∗^*p* < 0.05, ^∗∗^*p* < 0.01.

The migration of satellite cells to the injury site is a key process in the regeneration of skeletal muscles [25]. IGF1 was previously reported to involve in cell migration, as well as in skeletal muscle hypertrophy [26, 27]. Meanwhile, miR-204 has also been found to inhibit the migration of osteosarcoma [28]. Taken together, it is possible; therefore, we hypothesized that IGF1 and miR-204 are related to the myocyte migration. Interestingly, we found that the 3'UTR region of the *IGF1* has binding sites for miR-204 (Figure 3(a)). Further, we confirmed the binding of miR-204 to *IGF1* by a dual-luciferase reporter system (Figure 3(b)). The miR-204 mimic significantly inhibited the expression of *IGF1* and *Pax3*, which is the muscle satellite cell migration marker gene (Figure 3(c)). In addition, the scratch test showed that the miR-204 mimic significantly inhibited cell migration, while the miR-204 inhibitor significantly promoted cell migration (Figures 3(d) and 3(e)). All these results suggest that miR-204 may inhibit the migration of C2C12 myoblasts through *IGF1*.

### 3.3. Mef2c Is a Target Gene of miR-204 That Regulates the Differentiation of C2C12 Myoblasts

**Figure 4:**
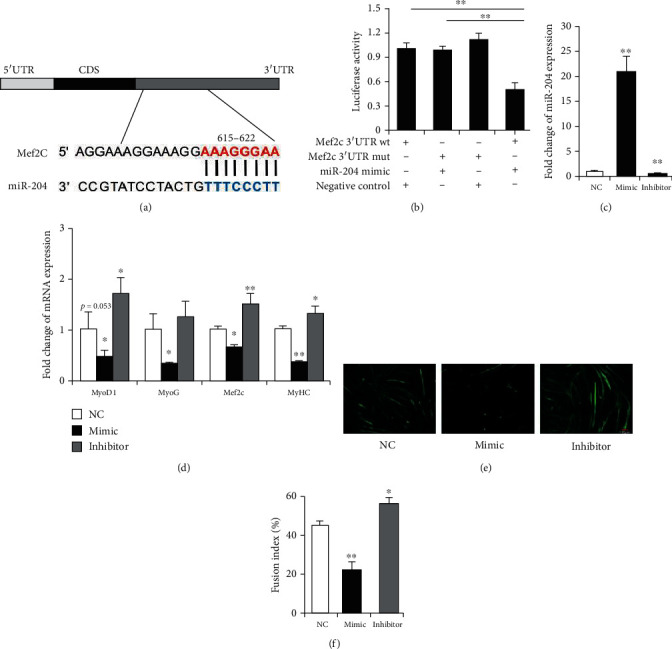
miR-204 regulates C2C12 myoblasts differentiation through *Mef2c* (myocyte enhancer factor 2c). (a) The binding sites of miR-204 to *Mef2c*. (b) The *Mef2c* 3'UTR with wild-type (wt) or mutant type (mut) were inserted into the psiCHECK™-2 vectors and cotransfected with miR-204 mimic or negative control into HeLa cells, respectively. *n* = 3. (c, d) The expression of miR-204, *MyoD*, *MyoG*, *Mef2c*, and *MyHC* in the C2C12 myotubes after transfection with miR-204 mimic or inhibitor. *n* = 3. (e, f) Immunofluorescence of MyHC (muscle myosin heavy chain) in C2C12 myotubes. Scale bar = 100*μ*m, *n* = 3. Data are presented as means ± SD. ^∗^*p* < 0.05, ^∗∗^*p* < 0.01.

Further, we found and confirmed the binding relationship between miR-204 and *Mef2c* (Figures 4(a) and 4(b)), which was reported to promote myoblasts differentiation. Compared with the negative control group, the expression level of miR-204 in the mimic group was increased by approximately 21-fold in C2C12 myotubes. However, the expression of miR-204 was down to 45% in the inhibitor group (Figure 4(c)). As shown in Figure 4(d), compared with the control group, the expression of *MyoG*, *Mef2c*, and *MyHC* in C2C12 myoblasts was significantly downregulated by the miR-204 mimic. On the contrary, the expression of *MyoD*, *Mef2c*, and *MyHC* was significantly increased after the miR-204 inhibitor transfection (Figure 4(d)). *MyHC* is a myogenic differentiation marker of myocytes, which was stained by MyHC antibody. The immunocytochemistry results showed that myocytes fusion rate was significantly decreased after the transfection of miR-204 mimic, whereas the fusion index was increased by transfection of miR-204 inhibitor (Figures 4(e) and 4(f)).

## 4. Discussion

**Figure 5:**
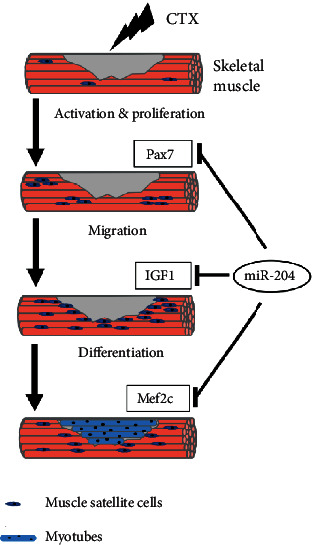
Schematic showing the regulation pattern of miR-204 on skeletal muscle. miR-204 can target *Pax7*, *IGF1*, and *Mef2c* in the process of myoblast proliferation, cell migration, and differentiation, respectively, and may contribute to the CTX-mediated mouse skeletal muscle injury.

In the present study, we found that miR-204 is downregulated during skeletal muscle regeneration *in vivo*. The dual-luciferase reporter system and qRT-PCR demonstrated that miR-204 can bind to the 3'UTR of *Pax7*, *IGF1*, and *Mef2c* and then inhibit their expression level. Cell proliferation assay, scratch assay, and differentiation assay showed that miR-204 can inhibit the proliferation, migration, and differentiation through its corresponding targets in C2C12 myoblasts, respectively (Figure 5).

*Pax7* is exclusively expressed in satellite cells and plays a crucial role in self-renewal and myogenic potential maintenance in adult skeletal muscle [29]. In the present study, it was found that the expression level of *Pax7* increased significantly on the 1st day after muscle injury, reached the highest level at 3d, and began to fall back after 7d. miR-204 exhibits an opposite expression pattern to *Pax7*. All these results suggest that miR-204 may also play a crucial role in skeletal muscle regeneration. Furthermore, the dual-luciferase reporter system confirmed the binding relationship between miR-204 and *Pax7*. In previous researches, miR-204 has been reported to have the ability to inhibit the proliferation of a variety of normal cells [30] and cancer cells [31]. In our study, we consistently found that miR-204 can inhibit the expression of *CCNB*, *CCND*, *CCNE*, and *CDK4*, which promote cell cycle progression [32, 33]. In addition, CCK8 and EdU assays showed that miR-204 mimic significantly inhibited cell proliferation while the miR-204 inhibitor significantly promoted cell proliferation.

*Pax3* and *IGF1* were found to be involved in cell migration [34, 35]. miR-204 has also been found to inhibit the migration of osteosarcoma [28]. Interestingly, we found and confirmed the binding relationship between miR-204 and *IGF1*. In this study, cell scratch assays also showed that miR-204 can inhibit the migration of C2C12 myoblasts. These results suggest that the role of miR-204 in inhibiting cell migration may exist among various types of cells.

When the satellite cells complete proliferation and migrate to the muscle injury site, the myocytes begin to differentiate and fuse with each other to form multinucleated myotubes [2, 36]. MRFs (myogenic regulatory factors) and *Mef2c* (myocyte enhancer factor 2c) have the ability to promote satellite cell differentiation, which were essential during the myogenesis process [22, 27]. During the differentiation of C2C12 myoblasts, miR-204 inhibited its target gene *Mef2c* and also decreased the expression level of *MyoD*, *MyoG*, and *MyHC*. And further immunofluorescence staining verified that miR-204 inhibited the myocyte differentiation through *Mef2c*.

## 5. Conclusion

In summary, we focused on the expression and function of miR-204 during skeletal muscle regeneration in the present study. The results suggest that the downregulation of miR-204 expression may be associated with skeletal muscle regeneration. Furthermore, our results show that inhibition of miR-204 promoted the expression of *Pax7*, *IGF1*, and *Mef2c*, which are the target genes of miR-204, and are also closely related to skeletal muscle regeneration. These results suggest that miR-204 may regulate the activation, proliferation, migration, and differentiation of myocytes through *Pax7*, *IGF1*, and *Mef2c*. Our findings reveal the potential of miR-204 in the treatment of skeletal muscle-related diseases.

## Figures and Tables

**Table 1:** The primers used for qRT-PCR and the synthesized RNA oligonucleotides.

Gene	Primer sequence (5′-3′)	TM/°C
*Pax3*	F-CCGGGGCAGAATTACCCAC	60.6
	R-GCCGTTGATAAATACTCCTCCG	
*Pax7*	F-TGGGGTCTTCATCAACGGTC	60
	R-ATCGGCACAGAATCTTGGAGA	
*IGF1*	F-GGACGGGGACTTCTGAGTCTT	60.6
	R-AGAGAGCGGGACTCCTTCTG	
*Mef2c*	F-ATCTCTCCCTGCCTTCTACTC	60
	R-CTCCCATCGTAGGAACTGCT	
*MyoG*	F-GCCCAGTGAATGCAACTCCCACA	58
	R-CAGCCGCGAGCAAATGATCTCCT	
*MyoD*	F-AGACTTCTATGATGACCCGTGTT	57
	R-TCAGCGTTGGTGGTCTTGC	
*MyHC*	F-GCGAATCGAGGCTCAGAACAA	60
	R-GTAGTTCCGCCTTCGGTCTTG	
*CCNB*	F-AAGGTGCCTGTGTGTGAACC	61
	R-GTCAGCCCCATCATCTGCG	
*CCND*	F-GCGTACCCTGACACCAATCTC	61
	R-CTCCTCTTCGCACTTCTGCTC	
*CCNE*	F-GTGGCTCCGACCTTTCAGTC	60
	R-CACAGTCTTGTCAATCTTGGCA	
*CDK4*	F-ATGGCTGCCACTCGATATGAA	60
	R-TCCTCCATTAGGAACTCTCACAC	
*β-Actin*	F-TGGAATCCTGTGGCATC CATGAAAC	60
	R-TAAAACGCAGCTCAG TAACAGTCCG	
*miR-204*	F-TTCCCTTTGTCATCCTATGCCT	60
	R- Uni-miR qPCR primer, included in kit (miRNA universal downstream primer, TaKaRa)	
*U6*	F: CTCGCTTCGGCAGCACA	60
	R: AACGCTTCACGAATTTGCGT	

F: forward; R: reverse.

## Data Availability

The data used to support the findings of this study are included in the article.
